# A sensitivity study of urbanization impacts on regional meteorology using a Bayesian functional analysis of variance

**DOI:** 10.1007/s00477-025-03032-x

**Published:** 2025-06-24

**Authors:** Giacomo Moraglia, Matthew Bonas, Paola Crippa

**Affiliations:** 1 Department of Civil and Environmental Engineering and Earth Sciences, University of Notre Dame du Lac, Notre Dame, IN 46556 USA; 2 Department of Applied and Computational Mathematics and Statistics, University of Notre Dame du Lac, Notre Dame, IN 46556 USA

**Keywords:** Urban rainfall effect, Functional ANOVA, Urban heat Island, WRF-Chem, Aerosols

## Abstract

Urbanization affects atmospheric boundary layer dynamics by altering cloud formation and precipitation patterns through the urban heat island (UHI) effect, perturbed wind flows, and urban aerosols, that overall contribute to the urban rainfall effect (URE). This study analyzes an ensemble of numerical simulations with the Weather Research and Forecasting (WRF) model and its version with coupled chemistry and aerosols (WRF-Chem) through a Functional ANalysis Of VAriance (FANOVA) approach to isolate the urban signature from the regional climatology and to investigate the relative contributions of various mechanisms and drivers to the URE. Different metropolitan areas across the United States are analyzed and their urban land cover and anthropogenic emissions are replaced with dominant land-use categories such as grasslands or croplands and biogenic only emissions, as in neighboring regions. Our findings indicate a significant role of the urban land cover in impacting surface temperature and turbulent kinetic energy over the city, and precipitation patterns, both within and downwind of the urban environment. Moreover, simulations of a deep convection event suggest that the aerosols impact dominates the sign and spatial extent of the changes in the simulated precipitation compared to the UHI effect, leading to a significant precipitation enhancement within the urban borders and suppression in downwind regions.

## Introduction

The Urban Heat Island (UHI) effect, characterized by significantly higher temperatures in urban areas compared to surrounding rural regions, is a major consequence of urbanization (Oke et al. 2017). This temperature increase is primarily driven by the reduced vegetation and the prevalence of heat-absorbing, low-albedo surfaces such as asphalt and concrete, which retain heat more effectively than natural landscapes. These horizontal and vertical thermal stress induced by urban environments can impact tropospheric circulations (Wang 2022), particularly by enhancing turbulent vertical motions within the Planetary Boundary Layer (PBL). This turbulence enhancement can alter both the intensity and spatial distribution of precipitation, affecting areas within and downwind of cities (Pathirana et al. 2014; Zhang et al. 2017; Moraglia et al. 2024). The influence of UHI on precipitation patterns is part of a broader phenomenon known as the Urban Rainfall Effect (URE), which refers to the modification of precipitation characteristics due to urbanization. Unique topographic features and different regional climates were found to impact both the UHI (Zhou et al. 2015; Manoli et al. 2019; Lin et al. 2024) and URE (Sui et al. 2024). Furthermore, it has been shown that the size of the urban area can have varying impacts on deep convection events, potentially leading to increased urban flooding and more severe extreme weather events when convection is intensified (Kingfield et al. 2017; Zhou et al. 2024). Not only the UHI contributes to the URE, but also urban aerosols are expected to play a significant role in altering precipitation features (Ramanathan et al. 2002; Bell et al. 2008; Cao et al. 2021). First of all, urban aerosols may alter the radiation balance by back-scattering solar radiation and thus reducing surface temperatures and stabilizing the atmosphere (Wang et al. 2024). Thus, aerosol may play an opposite role to the UHI that will tend to enhance turbulent mixing. Furthermore, urban aerosols, typically characterized by high number concentrations and small sizes, are known to suppress collision and coalescence during the early stages of deep convection (Givati and Rosenfeld 2004), while simultaneously enhancing cloud depth, which ultimately may lead to increased convective intensity and precipitation amounts (Han et al. 2012; Zhong et al. 2015; Fan et al. 2020). The impact of urban aerosols on the URE remains an area of active research. Large uncertainties exist regarding the extent, magnitude, and evolution of precipitation changes induced by aerosols, as well as on whether they primarily tend to enhance or suppress urban precipitation (Qian et al. 2022).

Numerical weather prediction and chemical transport models provide valuable tools for investigating the URE. Numerical experiments perturbing land-use properties and emissions allow to isolate the urban influence from the broader regional climatology, which is challenging when relying solely on observational data. For instance, previous studies examined the effects of land cover changes by conducting numerical experiments where urban areas are replaced with other land-use types (Zhong et al. 2016; Li et al. 2022; Zhou et al. 2024; Moraglia et al. 2024). However, as the output from these numerical models continues to increase in spatial and temporal resolutions, there is an increasing need for the development of statistical approaches to conduct comprehensive sensitivity analyses, particularly in light of the large uncertainty in the models’ response and ability to capture impacts of land surface processes (Pitman et al. 2009), aerosols and emission control policies (Georgescu et al. 2021) on weather and climate properties.

Classical statistical approaches, such as the widely used ANalysis Of VAriance (ANOVA) (Wilks 2011), allow us to analyze the sensitivity of a single variable to a finite number of factors. In its original formulation, ANOVA is not tailored for analyses where the variable is spatial (or spatio-temporal), as is typically the case in environmental applications. Indeed multiple, independent ANOVA for every location would not allow to borrow information for neighboring sites: the sensitivity of a particularly noisy location could still be assessed if spatially close locations are less noisy. Recent studies have shown that by integrating techniques from functional data analysis and spatial statistical modeling, the ANOVA methodology can be adapted for these spatial fields. Even in cases where the dimension in such settings is large, the parameter space remains manageable through a hierarchical approach and by accounting for the spatial dependencies in the data. This study proposes the use of Functional ANOVA (FANOVA), a generalization developed to perform sensitivity analyses over entire spatial or spatio-temporal output (Stone et al. 1997), which has successfully been implemented in many fields, including geoscience (Kaufman and Sain 2010; Sun and Genton 2012; Qu et al. 2021; Hobbs et al. 2024; Zhang et al. 2024).

In this work, we present a series of numerical simulations using the Weather Research and Forecasting model with coupled chemistry (WRF-Chem) aimed at quantifying the spatio-temporal variability induced by changes in urban land cover and urban emissions across various weather variables and time scales (e.g., long-term trends and an individual deep convection event). Specifically, this study has three main objectives: (i) to analyze the UHI and URE across different metropolitan areas in the United States on multiple time scales, (ii) to assess the impact of urban emissions on a deep convection event, and (iii) to propose a flexible statistical method for quantifying the significance of spatio-temporal changes in weather variables resulting from land-use changes.

The paper is structured as follows: Sect. 2 introduces the numerical simulations and Sect. 3 describes the statistical approach adopted. Section 4 is divided in two parts: we first present findings for the sensitivity analysis to land-use perturbations on temperature and turbulent kinetic energy with a one-way FANOVA, followed by results from a multi-way FANOVA designed to quantify sensitivity to both land-use and emission changes on precipitation patterns. We present concluding remarks in Sect. 5.

## Data

This work focuses on three large, densely populated, metropolitan areas across the United States, each characterized by a distinct regional climate and geographic features. The selected cities are Indianapolis, IN, which according with the Köppen classification is defined having a hot-summer humid continental climate (*Dfa*); the New York/Newark area, NY (henceforth NE corridor) and the Dallas-Fort Worth (DFW) Metroplex, TX, which both have a humid subtropical climate (*Cfa*). We adopt a model-based approach to be able to separate and analyze each distinct component of the URE. Specifically, a suite of numerical simulations is generated with the Weather Research and Forecasting model (WRF) (Skamarock et al. 2019) and its version with coupled chemistry (WRF-Chem) version v4.6.0 (Grell et al. 2005) to investigate the role of (i) land-use change on the UHI and (ii) anthropogenic and biogenic aerosols in impacting rainfall patterns. All simulations share identical physics schemes and spatial resolution, as summarized in Table 1, so discrepancies in model output can be attributed to the inclusion of aerosol coupling and land-use changes. Across all runs, the fifth-generation ECMWF atmospheric reanalysis ERA-5 (Hersbach et al. 2020), with a horizontal grid spacing of $0.25^{\circ }\,{\times }\,0.25^\circ$, provides hourly lateral boundary conditions. The land-use classification is obtained from the 2004 MODIS database at 900 m resolution and 21 land categories (Friedl et al. 2002; Hansen et al. 2002). Key physics parameterizations adopted are the Rapid Radiative Transfer Model for GCMs (RRTMG) scheme for the radiation budget (Iacono et al. 2008), the Mellor - Yamada - Nakanishi - Niin (MYNN) planetary boundary (Nakanishi and Niino 2009) and the Yonsei University (YSU) surface layer schemes (Hu et al. 2013). As for the microphsysics scheme, the Milbrandt-Yau double-moment scheme (Milbrandt and Yau 2005) is used for the WRF physics-only simulations, while the runs with WRF-Chem adopt the Morrison double-moment scheme, with ice-correction, as this microphysics scheme is dynamically coupled with the adopted aerosol module in WRF-Chem (Morrison et al. 2005). The runs including atmospheric chemistry and aerosols coupling with WRF-Chem use chemical boundary conditions from the Community Atmosphere Model with Chemistry (CAM-Chem) model (Lamarque et al. 2012), while the Model for Ozone and Related chemical Tracers (MOZART) for gas chemistry (Emmons et al. 2010) coupled with the Model for Simulating Aerosol Interactions and Chemistry (MOSAIC) sectional aerosol scheme (Zaveri et al. 2008) is adopted to simulate atmospheric chemistry and aerosol dynamics, respectively. Specifically, aerosols are categorized into four size bins: Bin1 (0.04−0.15 μm), Bin2 (0.15–0.63 μm), Bin3 (0.62–2.5 μm), and Bin4 (2.5–10 μm). Chemical option chem_opt = 202 is selected as it includes a large number of aqueous chemical reactions as well as key features as in-cloud chemistry and aerosol wet scavenging (Knote et al. 2014). This chemical scheme is paired with a full photolysis (TUV) (Madronich 1987). Anthropogenic emissions are provided by the U.S. Environmental Protection Agency National Emissions Inventory (NEI) (Pfister et al. 2011; Reff et al. 2020) with a 4 km horizontal resolution and hourly frequency, while biogenic emissions are derived from the Model of Emissions of Gases and Aerosols from Nature (MEGAN) (Guenther et al. 2012). Sea spray and dust are also included.

**Table 1:** Key physics and chemistry parameterizations adopted

Physics/Setup	Scheme/Value	Reference
Grid Spacing	12 km	–
Vertical Levels	61	–
Time step	30 sec	–
Microphysics (Long-term runs)	Millbrandt-Yau	Milbrandt and Yau (2005)
Microphysics (Deep convection)	Morrison	Morrison et al. (2005)
LW Radiation	RRTMG	Iacono et al. (2008)
SW Radiation	RRTMG	Iacono et al. (2008)
PBL	MYNN2	Nakanishi and Niino (2009)
Surface Layer	YSU	Hu et al. (2013)
Cumulus	Grell-Devenyi	Grell and Dévényi (2002)
Gas chemistry	MOZART	Emmons et al. 2010)
Aerosols	MOSAIC	Zaveri et al. (2008)
Chemical option	202	Knote et al. (2014)

**Fig. 1:**
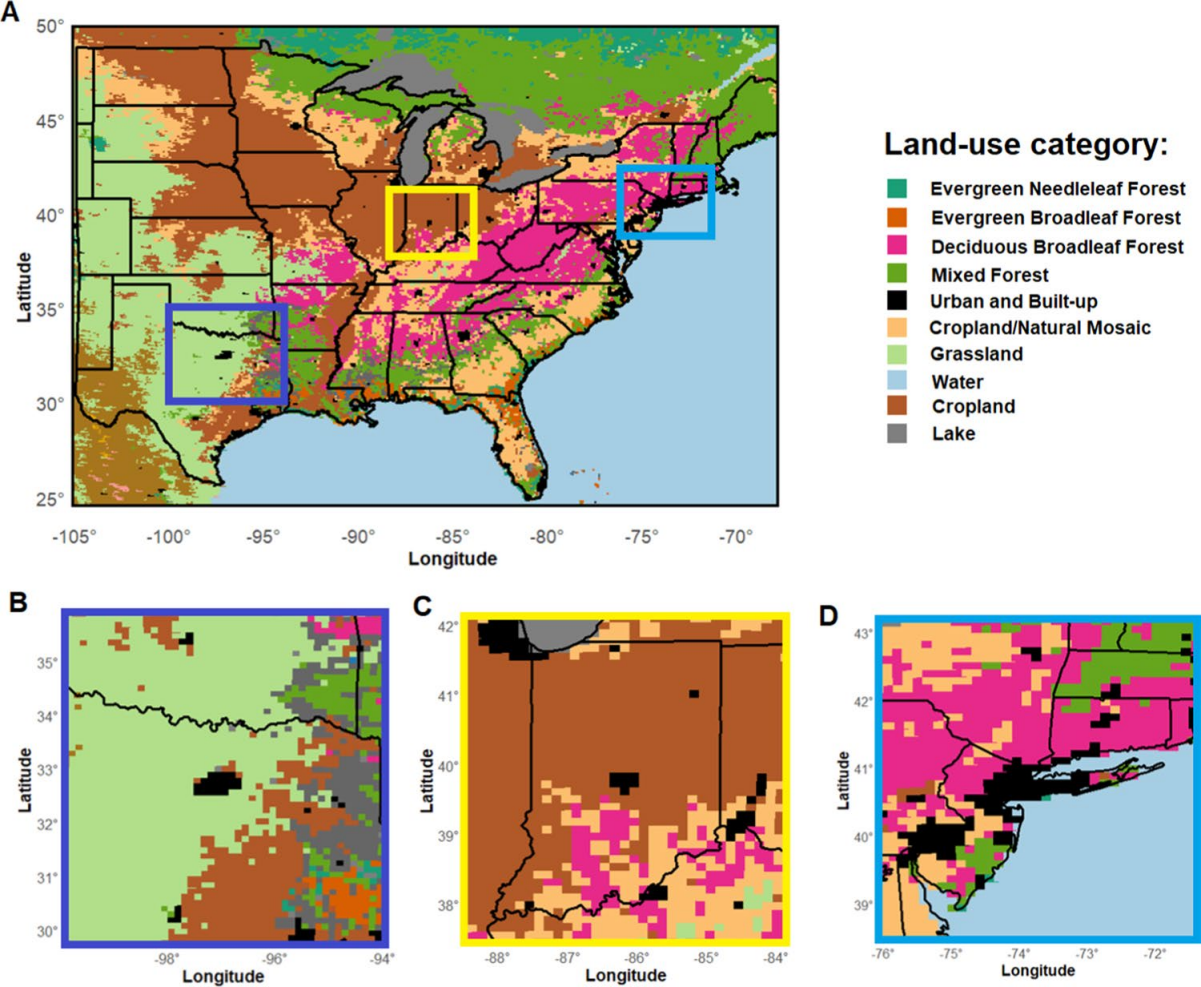
Simulated domains across the United States (**A**) for Dallas Fort-Worth (**B**) Indianapolis (**C**) and the North-East corridor (**D**) case studies. Most common MODIS 21 land-use categories in the analyzed domains are listed in the legend with respective colors

Indianapolis and the NE corridor will be used as case studies to investigate the model’s ability to capture persistent features associated with the land-use perturbation due to urbanization (UHI) over long time periods. These simulations extend over the two consecutive rainiest months, April–May 2017 for the NE corridor and the same months in 2018 for Indianapolis. The years 2017 and 2018 are chosen as climatologically representative for precipitation (i.e., they have the smallest yearly deviation from the 1991–2020 normal, based on the National Weather Service precipitation records (NWS 2023)). The focus on the rainiest months enables to investigate the UHI effect under a variety of weather events, thus contributing to the generalizability and robustness of our results. A set of perturbed simulations is generated for these two areas of interest to investigate the impact of land-use change on weather variables by replacing the “Urban and Built-Up” grid cells comprising Indianapolis and the NE corridor with “Croplands” and “Deciduous broadleaf forest", respectively, which correspond to the most common land-use category in the simulated areas surrounding each city. This is achieved by altering both the land-use index (LU_INDEX) and the land-use fraction (LANDUSEF) fields in WRF (Fig. 1B, C), similarly to what proposed in (Moraglia et al. 2024). The impact of land-use perturbation is investigated in a squared area of $\sim$200 km side from the center of city (Fig. 1A). This choice is motivated by previous studies showing that the impact of urbanization can be detected within this distance (Shepherd and Burian 2003; Liu and Niyogi 2019; Moraglia et al. 2024). Key variables analyzed are daily average 2 m temperature (T2) and near surface Turbulent Kinetic Energy (TKE).

Conversely, the simulations over DFW (Fig. 1B) are designed to capture a single deep convection event, that prior studies focused on to assess the model sensitivity to microphysics schemes and different boundary conditions in capturing the event’s characteristics (Zhou et al. 2024). The deep convection event was driven by a westerly mesoscale sized cold front reaching DFW on the 29th of March 2017. The simulations start 48 h before the day of the event occurrence and they end at 23:50 local time on the 29th, so the total simulated time period is from March 26th at 00:00 local time to March 29th at 23:50 local time. We only consider the 6 time points between 4:20 local time and 5:20 local time since this is the time lapse when the system exhibits the highest the reflectivity values (>55 dBz, not shown) while passing above DFW and in the downwind of the city. During this period the system produced relatively high amount of simulated rainfall ($\sim$45 mm in a single hour in the area). An ensemble of four runs is performed for this case study as follows:

(i) “City+No Emission": a physics-only run where the city is present, but emissions are not included, thus neither atmospheric chemistry or aerosols treatment is included.
(ii) “City+Emissions": a WRF-Chem run with full chemistry and aerosols coupling that includes the urban area of DFW.
(iii) “No city+Emissions": the “Urban and Built-Up" grid cells over DFW are replaced by “Grasslands" (i.e., the most abundant land-use category surrounding the city, Fig. 1B) and only biogenic emissions are included.
(iv) “No city+No Emissions": a phyiscs-only run where DFW “Urban and Built-Up" grid cells are replaced by “Grasslands" and no chemistry and aerosol treatment is included.

## Methods

Throughout this Section we introduce the FANOVA used to assess the effects of land-use and urban emissions to the atmospheric variables of the WRF model output. We mathematically define FANOVA in Sect. 3.1 and detail the Bayesian inference approach in Sect. 3.2.

### Functional ANOVA

Let *N* denote the total number of spatial locations. The random vector $\textbf{Y}_i(t) = \left( Y_{i}(\textbf{s}_{1}, t), \dots, Y_{i}(\textbf{s}_{N}, t)\right) ^{\top }$ represents the variable of interest at locations $\textbf{s}_{1}, \dots, \textbf{s}_{N}$ for some time point *t*. For the ease of notation, we assume that there is only one factor *i* which only has two levels $i \in \{0,1\}$: extensions to more then two levels or multiple factors are straightforward but are not detailed. FANOVA assumes that the data $\textbf{Y}_i(t)$ can be modeled as Zhang et al. (2024):

1a $$\begin{aligned}&\textbf{Y}_{i}(t) \thicksim \mathcal {N}\left( \varvec{\mu }_{i}(t),\sigma ^2\right) \end{aligned}$$

1b $$\begin{aligned}&\varvec{\mu }_{i}(t) = \textbf{f}(t) + i\varvec{\beta } \end{aligned}$$

1c $$\begin{aligned}&\textbf{f}(t) = \sum _{k=1}^{K}\left\{ \varvec{\alpha }_{k}\text {sin}\left( \frac{2\pi kt}{\delta } \right) + \varvec{\gamma }_{k}\text {cos}\left( \frac{2\pi kt}{\delta } \right) \right\}, \end{aligned}$$

where the bold notation indicates a vector containing all *N* spatial locations. The random vector $\textbf{Y}_{i}(t)$ is drawn from a Normal distribution with mean $\varvec{\mu }_{i}(t)$ and variance $\sigma ^2$. For all spatial locations, the mean assumes a temporal effect $\textbf{f}(t)$ controlled by *K* harmonics with period $\delta$ for each $n = 1,\dots,N$ location independently, so that the total number of temporal parameters is $\varvec{\theta }_{\text {time}} = \{\varvec{\alpha }_{k}, \varvec{\gamma }_{k}; k = 1,\dots,K\}$. The key component of FANOVA is the latent spatial process $\varvec{\beta }$, which encodes the contribution of the factor *i* and is assumed to be Gaussian.

In a traditional ANOVA, one would analyze the significance of $\varvec{\beta }$ separately for every location, i.e., assuming independence in space. FANOVA, however, assumes that this random effect is dependent in space (yet still independent in time) according to a Gaussian random field:

2 $$\begin{aligned} \varvec{\beta } \thicksim \mathcal {N}(\textbf{0}, \varvec{\Sigma }) \end{aligned}$$

In this work, we use the Matérn function with smoothness 1, a widely used choice for modeling spatial dependence (Stein 1999), which assumes that the $jj'$th element of the covariance matrix $\varvec{\Sigma }$ is:

3 $$\begin{aligned} (\varvec{\Sigma })_{jj'} = \frac{\kappa }{\tau 2^{\nu -1}\Gamma (\nu )}\Vert \textbf{s}_{j} - \textbf{s}_{j'}\Vert K_{1}(\kappa \Vert \textbf{s}_{j} - \textbf{s}_{j'}\Vert ), \end{aligned}$$

where the parameter $\tau$ represents the marginal precision and $\kappa$ represents the range and describes the rate of decay of spatial correlation as a function of distance. The function $K_{1}(\cdot )$ is a modified Bessel function of second kind of order 1 and $\Vert \textbf{s}_{j}-\textbf{s}_{j'}\Vert$ is the Euclidean distance between two locations $\textbf{s}_{j}$ and $\textbf{s}_{j'}$.

In this work we model the Matérn covariance function by solving a stochastic partial differential equation (SPDE). This approach is widely used in spatial statistics (Zhang et al. 2024), and allows for computationally efficient inference. A Gaussian process with a Matérn covariance is a unique stationary solution of a fractional reaction-diffusion SPDE defined as Whittle (1954, 1963):

4 $$\begin{aligned} (\kappa ^2 - \Delta )(\tau \varvec{\beta }) = \mathcal {W}(\textbf{s}), \end{aligned}$$

where $\Delta$ represents the Laplacian operator and $\mathcal {W}(\textbf{s})$ is a spatially distributed Gaussian white noise with unit variance. We assume $\varvec{\beta }$ is a solution of Eq. (4), so that the spatial parameters are $\varvec{\theta }_{\text {space}} = (\kappa ^2, \tau )$. Both parameters are assumed to have independent vague $\mathcal {N}(0,1000)$ priors.

### Inference

Inference is achieved in two steps to reduce the computational burden of estimating all parameters at once. We first estimate the parameters of the temporal structure $\textbf{f}(t)$ from Eqs. (1a, 1b, 1c) independently for each spatial location. Subsequently, we fix these parameters to their posterior mean and perform inference on $\varvec{\theta }_{\text {space}}$. In order words, we perform inference on the parameters associated with the spatial dependence, conditional on the temporal structure.

While this two-step approach allows for considerable computational savings, spatial inference is still extremely demanding owing to the large number of spatial locations. To further mitigate this, we perform inference for the model in Eq. (1a, 1b, 1c) using an integrated nested Laplace approximation (INLA) approach (Rue et al. 2009; Bakka et al. 2018). This method allows fast approximation of high dimensional integrals in FANOVA and more generally latent Gaussian models.

### Model evaluation

The simulation over Indianapolis has been thoroughly evaluated in (Moraglia et al. 2024). Similar model performance has been observed for the NE Corridor run, as both simulations share an identical setup (mean bias $\sim$ +0.7 °C for T2 and $\sim$ + 1.7 mm for daily precipitation amounts averaged over the inner domain, not shown). Therefore, we focus our model evaluation on the DFW simulations, analyzing two key variables: 2-meter air temperature (T2), which serves as a metric for quantifying the urban heat island (UHI) effect, and precipitation amounts.

For T2, we compute the mean daily bias between model output and observations from 15 stations in the Global Historical Climatology Network (GHCN) (Menne et al. 2012) during March 26–28, 2017.

To evaluate precipitation amounts, we compare model output with hourly observations from NEXRAD Stage IV (Du 2011). RADAR observations are bi-linearly interpolated to align with the simulation grid (i.e., upscaled from the original 4 km resolution to dx = 12 km). The differences between model output and RADAR data are computed at 05:00 local time, corresponding to the peak intensity of the system over DFW, as described in Sect. 2.

## Results

### Temperature and turbulence sensitivity to land-use

In this section we use FANOVA to estimate how the urban land-use can affect T2 and TKE, both of which are key indicators of the UHI effect, based on long-term WRF simulations centered over Indianapolis and the NE corridor (which includes the metropolitan areas of New York City, NY and Philadelphia, PA). We assess the sensitivity of these environmental variables to land-use changes associated with urbanization using a one-way FANOVA setting as defined in Sect. 3.1. We choose $K=3$ harmonics with an annual period for the mean of the FANOVA in Eq. (1c). FANOVA is applied over the areas of interest in order to analyze whether the urban land-use would have any impact on the response variables. For the ease of reading, in the FANOVA in Eq. (1b) we denote the effect of urban land-use as $\varvec{\beta }_{\text {city}}$ (instead of $\varvec{\beta }$), so that a positive value can be interpreted as that there is an increase in T2 and TKE due to the presence of the urban land-use compared to croplands since in variable $\varvec{i}$ in this same equation denotes city presence in the simulation. Additionally, the variables of interest (T2 and TKE) are to be consider the variable $\textbf{Y}_{\varvec{i}}(t)$ in Eq. (1a). Formally, we can rewrite and consider Eqs. (1a) and (1b) as:

5a $$\begin{aligned}&\textbf{TKE}_{i}(t) \thicksim \mathcal {N}\left( \varvec{\mu }_{i}(t),\sigma ^2\right) \end{aligned}$$

5b $$\begin{aligned}&\varvec{\mu }_{i}(t) = \textbf{f}(t) + i\varvec{\beta }_{\text {city}}. \end{aligned}$$

**Fig. 2:**
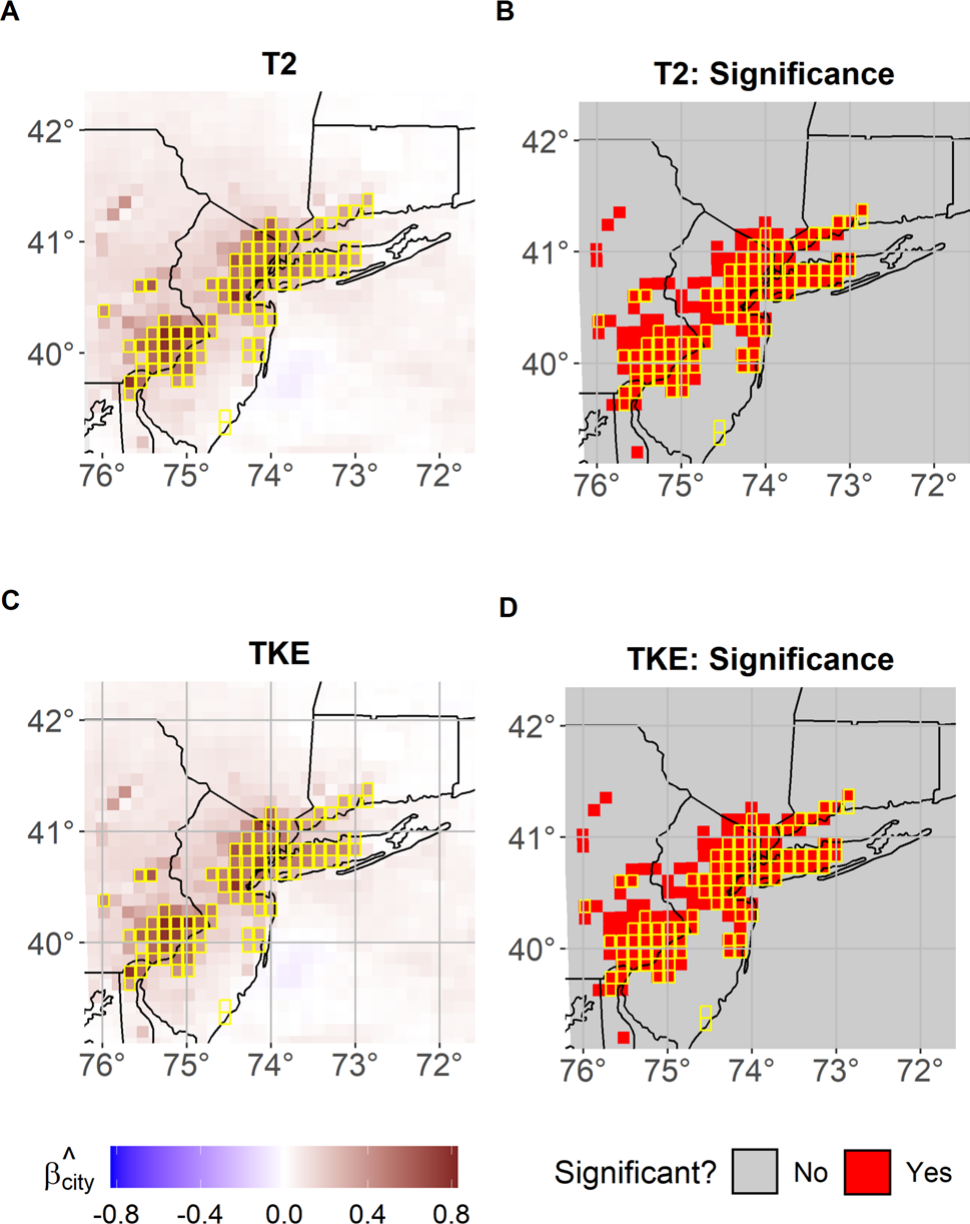
FANOVA model as in (1a, 1b, 1c) for T2 and TKE in the NE corridor. Panels A and C show the estimated posterior mean of $\varvec{\beta }_{\text {city}}$ for T2 and TKE, respectively. Panels B and D maps the locations whose 95% credibility interval for the posterior of $\varvec{\beta }_{\text {city}}$ do not contain the value of 0 for T2 and TKE, respectively. Contoured in yellow the urban grid cells replaced with “Deciduous broadleaf forest" land use category. Analyzed time period is April–May 2017

Figure 2 depicts the estimated posterior mean of $\varvec{\beta }_{\text {city}}$ for both T2 and TKE in panels A and C, respectively, for the NE corridor. The estimated coefficients are large and positive over the entire region, indicating that the presence of the urban land-use increases substantially T2 and TKE. At a maximum, the presence of the city would increase T2 and TKE by $0.83 ^{\circ }$C and 0.5 m^2^s^−2^, respectively. While prior studies have shown that the UHI may exhibit peaks ranging from +1 °C to +4 °C (Arnfield 2003; Yin et al. 2023), especially during nighttime, our focus on daily mean T2, as well as on the rainiest months during the spring season, inevitably reduces its magnitude. Figure 2 B and D indicate that the metropolitan area has a substantial effect on both the T2 and TKE. The significance of a location is identified based on the 95% credibility interval for the posterior distribution of $\varvec{\beta }_{\text {city}}$: a location is deemed significant if this interval does not contain the value of 0. From these results it is readily apparent that not only does the UHI have a significant effect on both T2 and TKE, but also the FANOVA approach is able to extract this effect while maintaining spatial coherence. If a standard ANOVA approach were to be considered, one would have to conduct the analysis independently across spatial locations. This would then neglect any potential spatial dependence structure present, which ultimately is a large advantage of FANOVA approach as shown by these results. Additionally, using an ANOVA independently across spatial locations would then require discussion on the family-wise error (FWE) rate. Conducting an independent ANOVA for each spatial location would naturally incur an inflated FWE which would ultimately need correcting via methods like False Discovery Rate (FDR) or Bonferroni, which is beyond the scope of this work.

**Fig. 3:**
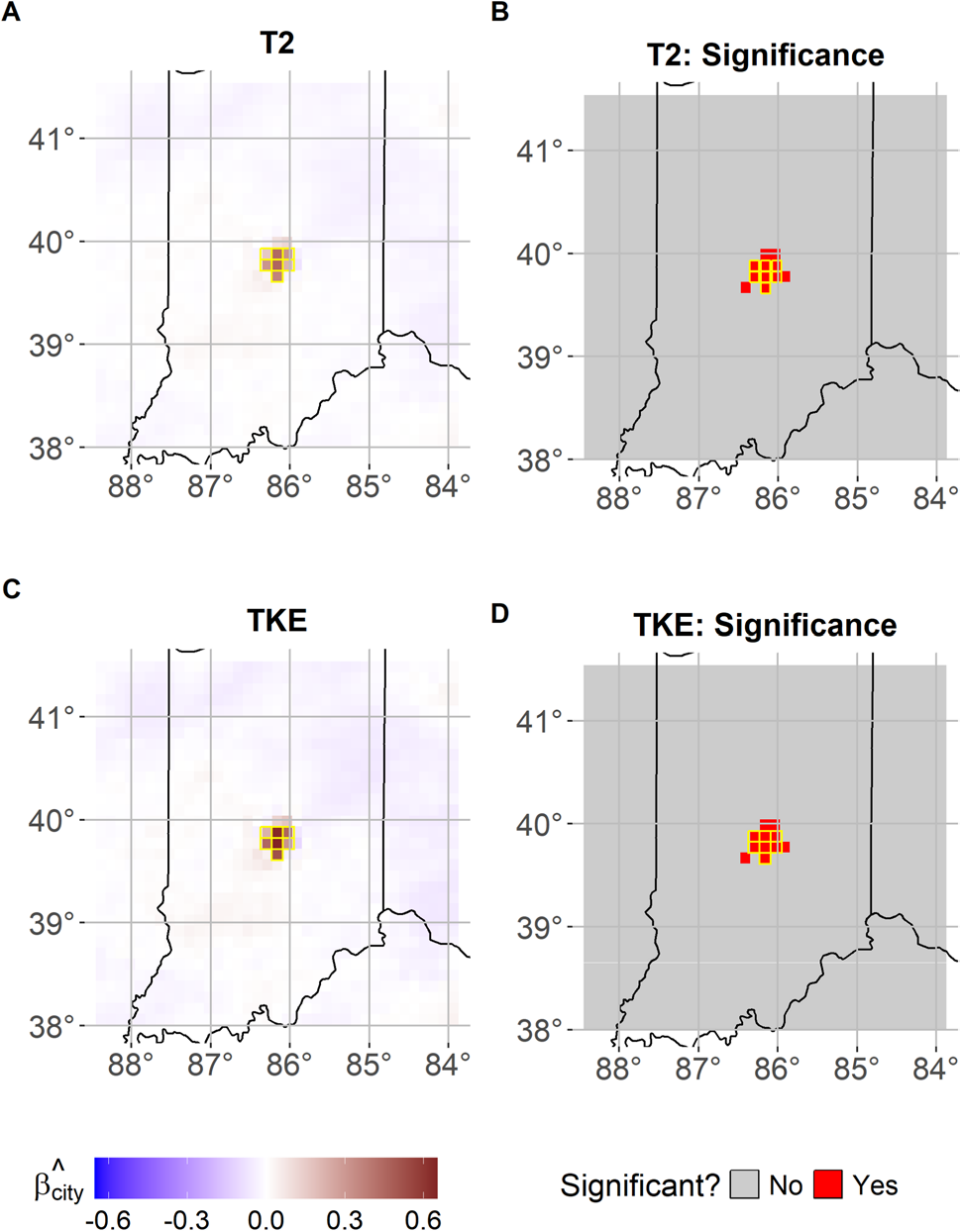
FANOVA model as in (1a, 1b, 1c) for T2 and TKE in the region near Indianapolis, IN. Panels A and C show the estimated posterior mean of $\varvec{\beta }_{\text {city}}$ for T2 and TKE, respectively. Panels B and D maps the locations whose 95% credibility interval for the posterior of $\varvec{\beta }_{\text {city}}$ do not contain the value of 0 for T2 and TKE, respectively. Contoured in yellow the urban grid cells replaced with “Croplands" land use category. Analyzed time period is April–May 2018

In the case of the area surrounding Indianapolis, Fig. 3A and C show the estimated posterior mean of $\varvec{\beta }_{\text {city}}$ for both T2 and TKE, respectively. Unlike the case of the NE corridor, this region does not have a large urban composition and comprises of a majority of non-urban land-use types, such as croplands. Despite this vastly different composition and the much smaller spatial extent of the urban area than the NE corridor analysis, the FANOVA model is still able to extract coefficient estimates with large positive values over the comparatively smaller urban region. For this case, at a maximum, the presence of the city would increase T2 or TKE by 0.45 °C and 0.65 m^2^s^−2^, respectively. The significance of $\varvec{\beta }_{\text {city}}$ from the 95% credibility intervals generated for both T2 and TKE are shown in panels B and D, respectively. As before, these panels show the spatial locations whose estimated credibility intervals do not contain the value of 0. Even though the majority of the domain is classified as non-urban, FANOVA still has the ability to extract the significant effect over the city. In this case, an approach such as ANOVA would be inappropriate since the spatial dependence would be discarded. Additionally, since these ANOVA tests would be conducted independently, one would have to control for the increased risk of making a type I error when conducting such a large number of tests. As stated previously, this would require correction via standard approaches such as False Discovery Rate (FDR) or Bonferroni and the choice and discussion around this correction is beyond the scope of this work. The FANOVA approach accounts for both these issues directly by modeling the spatial dependence structure of the data while conducting the statistical test.

From a physical perspective these results indicate that, for the NE corridor, the energy surplus produced by the UHI effect is associated with a significant warming not only over the urban grid cells but also in neighboring ones outside the urban areas. Conversely, for TKE (Fig. 2D), significant discrepancies are identified only over the urban grid cells (Fig. 1D). This can be explained by considering that changes in TKE primarily reflect changes in the surface roughness, which influence vertical turbulent fluxes. As expected, when the urban land cover is replaced by other land-use categories with lower surface roughness (e.g., deciduous broadleaf forest or croplands), all of the former urban grid cells show significant a increase in TKE. As for T2, Fig. 2A-B indicates instead that several rural grid cells surrounding the major cities of Philadelphia and NY also show significant increase in temperature. This is likely due to the horizontal fluxes and advection of heat which propagate to neighboring cells from the urban ones (Fig. 1D). Interestingly, this feature does not emerge in the Indianapolis case, probably due to its smaller spatial extent. In that case, the changes in T2 and TKE are solely confined within the city boarders (Fig. 1C) which coincide again where the change in TKE is significant. Overall, our findings are consistent with previous work by Zhou et al. (2015) that indicated that the UHI effect could manifest over spatial extent up to 3.9 times of urban size and with Manoli et al. (2019) who showed that the magnitude of the UHI is generally proportional to urban city size and population.

### Precipitation sensitivity to land-use and aerosols

**Fig. 4:**
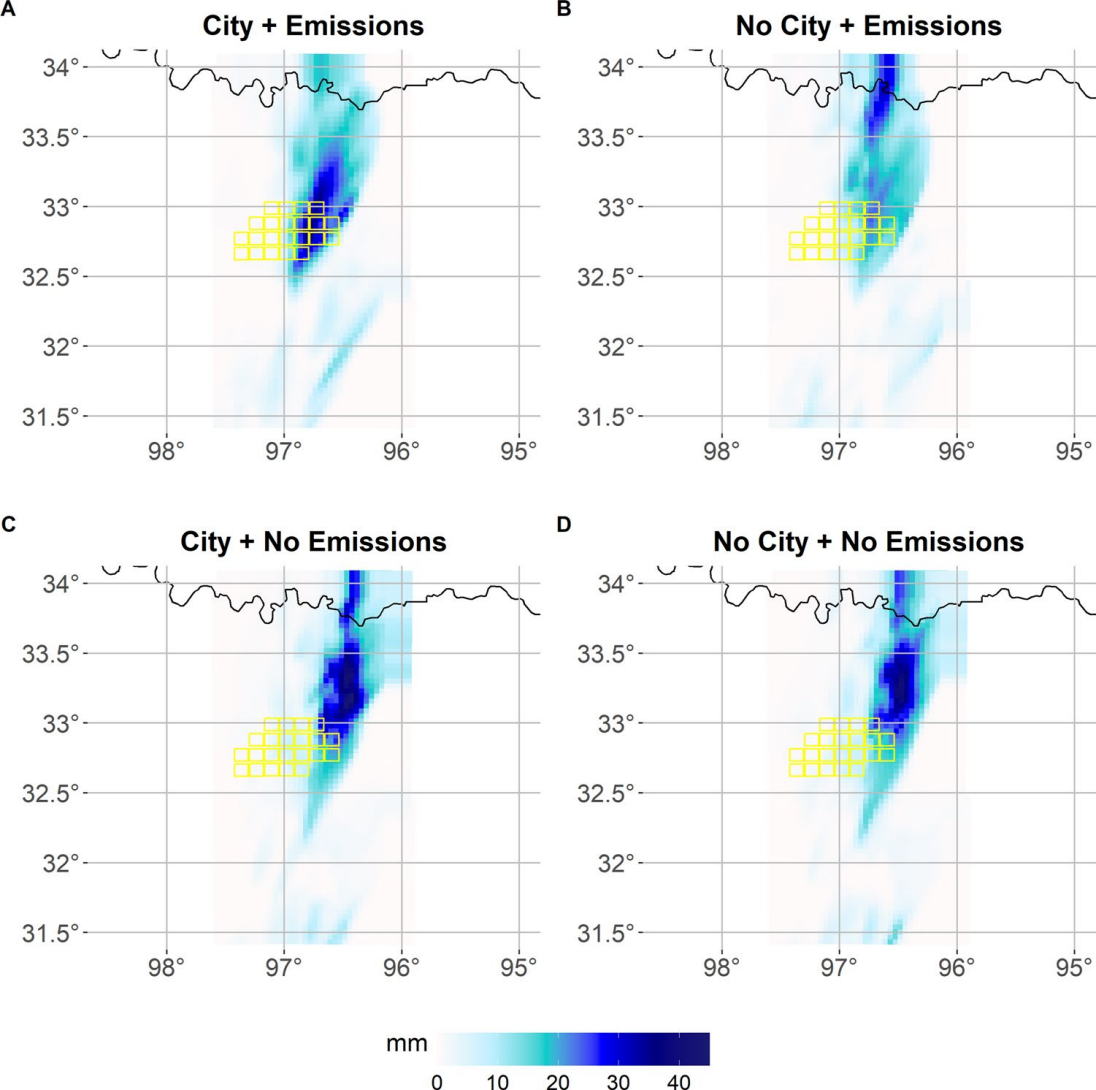
Total precipitation amounts [mm] simulated between 4:20 local time and 5:20 local time (i.e, while the system exhibits the highest values of reflectivity (not shown) while over-passing DFW and in the downwind of the city) for the four simulations analyzed for the deep convection event (as described in Sect. 2)

In this section we quantify the sensitivity of precipitation to land-use changes and aerosols presence over and surrounding the metropolitan area of DFW using FANOVA. As explained in detail in Sect. 2, in this analysis we employ WRF-Chem and we focus on the time period when the system has reached downwind areas of the city and shows the highest reflectivity. Thus any discrepancy in the simulated precipitation across model runs can be causally linked to perturbations driven by the system over-passing the DFW area. DFW is situated in the well-known "flash-flood alley" (Saharia et al. 2017), which has historically seen multiple fatalities due to extreme precipitation from deep convective events (Sharif et al. 2015). A comparable event is analyzed here: Fig. 4 illustrates peaks of accumulated precipitation reaching up to 45 mm within a single hour in the area. Being able to accurately forecast these deep convection events is therefore crucial for public safety.

Both the City+Emissions and City+No Emissions simulations exhibit a similar bias in T2, with the model consistently overestimating this variable. The bias, calculated for March 26–28, 2017, remains small across both urban and rural areas when compared with observations from 15 GHCN stations. Specifically, the T2 bias ranges from +0.5 to +1.0$^\circ$C, with no significant differences between the two simulations. Additionally, no significant discrepancies are observed between urban and rural biases (not shown). These findings align with previous research (Crippa et al. 2019).

**Fig. 5:**
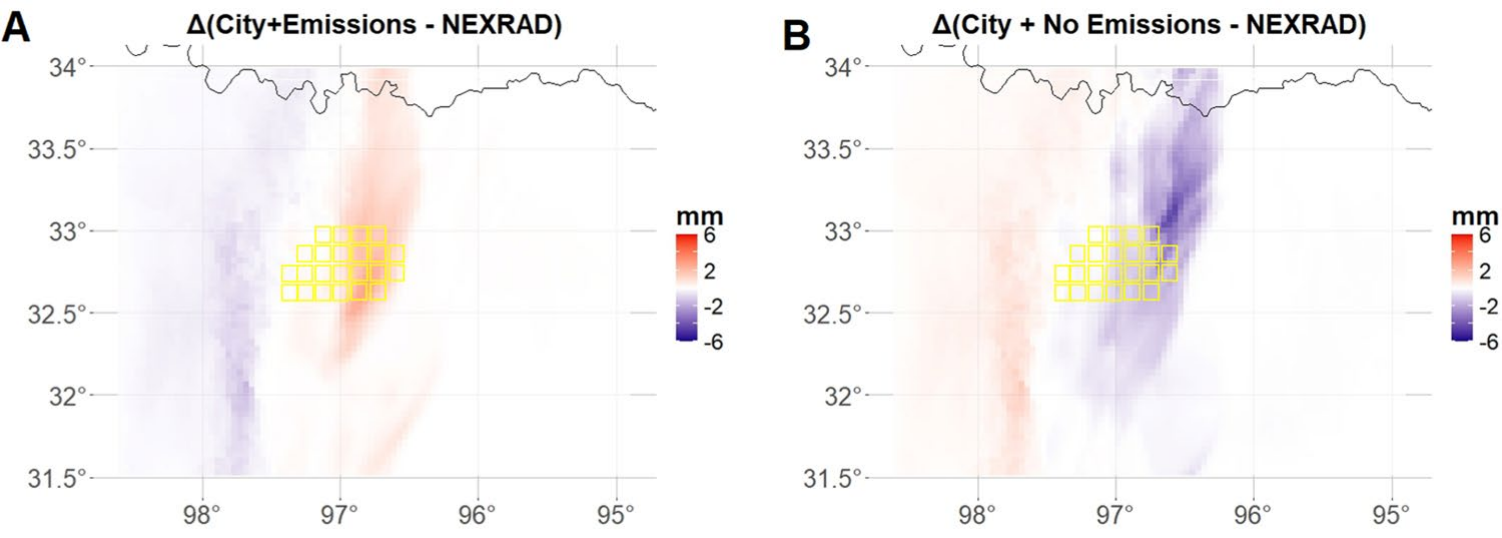
Difference in precipitation (mm) between City+Emissions (City+No Emissions) simulations and NEXRAD Stage IV hourly observations, calculated on the total hourly precipitation at the time of the peak (i.e., 4:00–5:00 local time)

In contrast, precipitation predictions exhibit more pronounced discrepancies. During the peak period analyzed, the City+Emissions simulation shows smaller differences compared to NEXRAD Stage IV observations, while the City+No Emissions simulation significantly underestimates precipitation by up to $-6$ mm, particularly over the DFW urban area. Conversely, the City+Emissions run slightly overestimates precipitation ($\sim$ +2 mm) in the same urban grid cells but demonstrates overall higher accuracy (Fig. 5).

Given the highly localized and short temporal range of the precipitation event analyzed, unlike the case in Sect. 4.1, we do not assume any change in the temporal trend of precipitation through annual harmonics. To model the temporal structure of the data, we instead consider each six 10-minute observations as replicate. As in the UHI case, for ease of reading, in the FANOVA in Eq. (1b), we denote the effect of urban land use as $\varvec{\beta }_{\text {city}}$ (instead of $\varvec{\beta }$), so that a positive value assesses the added precipitation in response to urban land use. Furthermore, we denote by $\varvec{\beta }_{\text {emiss}}$ the effect of emissions where a positive value is associated with an increase in precipitation due to the inclusion of emissions as described in Sect. 2. Furthermore, similar to what was noted in Sect. 4.1, the variable of interest in this case, precipitation (PRCP), is considered to be $\textbf{Y}_{\varvec{i}}(t)$ in the Eq. (1a). This case considers a two-level FANOVA model where $\varvec{i}$ in Eq. (1b) represents the indicator of urban land use and another indicator $\varvec{j}$ which denotes the indicator for consideration of emissions. Formally, we can rewrite and consider Eqs. (1a) and (1b) as:

6a $$\begin{aligned}&\textbf{PRCP}_{i,j}(t) \thicksim \mathcal {N}\left( \varvec{\mu }_{i,j}(t),\sigma ^2\right) \end{aligned}$$

6b $$\begin{aligned}&\varvec{\mu }_{i,j}(t) = \textbf{f}(t) + i\varvec{\beta }_{\text {city}} + j\varvec{\beta }_{\text {emiss}}. \end{aligned}$$

**Fig. 6:**
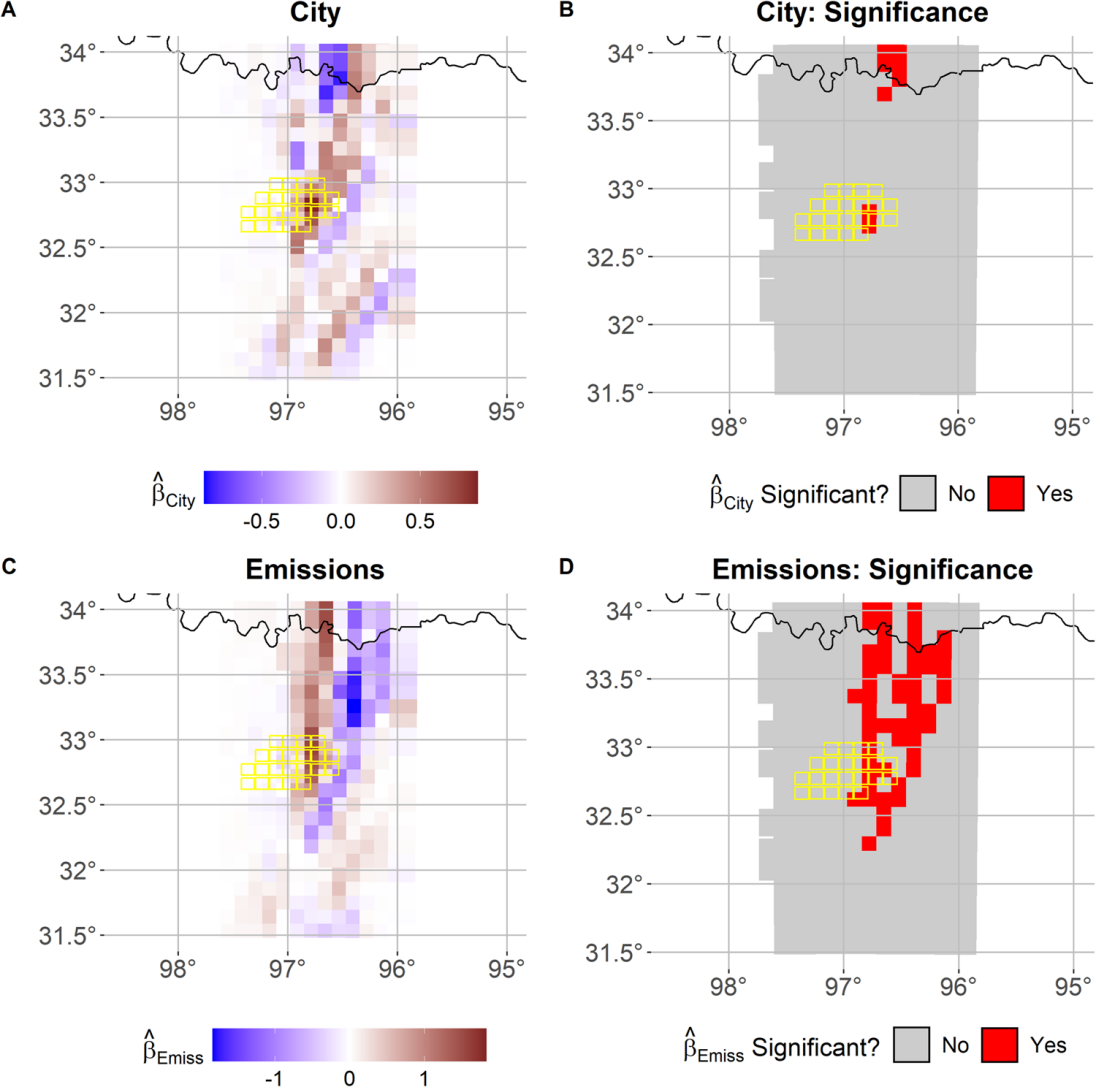
FANOVA results for precipitation in the region over Dallas-Fort Worth Metroplex, TX, USA. Panels A and C show the estimated posterior mean of $\varvec{\beta }_{\text {city}}$ and $\varvec{\beta }_{\text {emiss}}$, respectively. Panels B and D depict the spatial locations whose 95% credibility interval for the estimated coefficient do not contain the value of 0, meaning they are statistically significant for the effects from the city and emissions, respectively. Contoured in yellow DFW urban grid cells. Analyzed period is between 4:20 and 5:20 local time

Figure 6 shows the estimated posterior mean of $\varvec{\beta }_{\text {city}}$ and $\varvec{\beta }_{\text {emiss}}$ in panels A and C, respectively, for the DFW region. Both the land-use and emission effects are very large and positive values over the metropolitan area at the center of the domain, while the emission effect is large and negative in downwind regions, East-North-East of the city. In fact, Fig. 6A indicates that the presence of urban land use increases the amount of precipitation in the urban grid cells and neighboring ones in the downwind. Panel C instead indicates that the presence of emissions leads to increased precipitation directly over the urban environment, but also leads to a decreased amount in the downwind of the urban areas. This is likely due to high concentrations of cloud condensations nuclei originating from urban aerosols (Zhong et al. 2015; Fan et al. 2020): after deep convective clouds develop, the presence of high aerosol concentrations, even if small in size, can potentially lead to the development of a stronger convection. Indeed, (Han et al. 2012) showed with a different numerical model how the increased release of latent, heat resulting from the enhanced condensation process with increasing aerosol concentration, can lead to enhancement precipitation after a first stage of suppression (Fan et al. 2018). The model exhibits a significant difference at the Oklahoma-Texas border. Figure 4 illustrates that when aerosols are included in the simulation, the precipitation peak occurs primarily over the city. In contrast, in the No Emissions case, the peak shifts downwind to the northeastern part of the domain. This shift is driven by the highest aerosol concentrations in the City+Emissions scenario, which enhance precipitation over the urban area while reducing amounts downwind. These findings align with previous research demonstrating how urban aerosols can influence precipitation patterns in a city’s downwind region (Van Den Heever and Cotton 2007; Zhong et al. 2015).

Furthermore, our approach also allows us to quantify the relative magnitude of impact of the two drivers. Specifically, the presence of the city is found to increase the 10-minute time step precipitation over the city by a maximum of 0.9 mm (Fig. 6A), while the presence of urban emissions and associated aerosols increase the 10-min time-step precipitation by a maximum of ~1.8 mm over the city and decrease it by the same amount downwind (Fig. 6C). The significance of the effects from the 95% credibility intervals for the urban land-use and aerosols effects is shown in Fig. 6B and D, respectively. As in Sect. 4.1, these panels depict the spatial locations whose estimated credibility intervals for the coefficient estimate do not contain the value of 0. From Fig. 6B it is clear that the urban land cover in this region has a significant effect on hourly precipitation directly over the city. Additionally, Fig. 6D indicates that the aerosol coupling has a significant large effect on the simulated precipitation amounts both over the city and downwind from DFW. FANOVA is therefore able to detect a maximum increase of $\sim 2.7$ mm of precipitation amounts over the urban area when aerosols are included in the modeling process. In this application, the standard ANOVA approach would again need to be conducted independently across spatial locations and in this instance the spatial dependence is especially imperative to understanding the precipitation process. Given the lack of inclusion of the dependence directly in the model, ANOVA would not be appropriate to implement in this scenario, hence the necessity of a model like that of the proposed FANOVA in this work.

## Conclusions

This study aims to quantify the impact of urbanization on key meteorological variables using numerical simulations with the WRF model and its version with coupled chemistry, WRF-Chem. These simulations investigate how changes in land use influence the effect of UHI and the role of both anthropogenic and biogenic aerosols in the modification of rainfall patterns. To perform sensitivity analysis, we employ FANOVA on spatio-temporal output of the ensemble runs. Accounting for spatio-temporal dependence is crucial to accurately capture the UHI effect and changes in precipitation patterns discussed throughout this work.

In the first part of our analysis we show how perturbing the land-use (i.e., replacing urban grid cells with cropland and broadleaf forest) leads, as expected, to statistically significant changes in surface temperature but also in TKE, which is consistent with prior literature (Moraglia et al. 2024). The Indianapolis UHI effect is confined to the urban area, while simulations over the NE corridor indicate rural areas surrounding the large conurbations of Philadelphia and New York also exhibit higher near surface temperatures. The FANOVA allows us to capture the spatial dependence and our findings are consistent with previous literature showing that the UHI is noticeable for large cities even outside the urban environment (Zhou et al. 2015) and that its magnitude is proportional to city’s extent and population density (Manoli et al. 2019). In the second part of our analysis, we investigate the complex interactions between the UHI effect and urban aerosols on precipitation patterns. Consistent with prior studies (e.g., Pathirana et al. 2014; Fan et al. 2020; Li et al. 2022), our results show that the UHI induces statistical significant changes in accumulated precipitation and its spatial distribution. However, aerosols are found to impact precipitation over larger spatial scales, extending beyond the urban grid cells. FANOVA results indicate that aerosols increase the magnitude of precipitation changes by 1–1.5 times, on average, compared to the UHI alone. Additionally, simulations including aerosol treatment show a spread of precipitation to areas downwind of the city. This suggests aerosols have a broader regional influence compared to land-use changes. For the analyzed deep convection event, the sensitivity to land-use and aerosols’ inclusion in the model does not manifest only in the spatial patterns, but also impacts the evolution of the storm as it passes over the urban area. Classical statistical approaches, such as ANOVA, fail to capture these dynamics as they do not account for the complex spatial structures inherent in the data, which are critical for understanding localized and evolving weather phenomena.

While this study combines unique chemical transport model simulations with advanced statistical approaches to investigate key weather processes of societal relevance, we acknowledge that a comprehensive assessment of aerosols’ long-term impacts on precipitation and extreme events cannot be draw by focusing on a single deep convection event. Nevertheless, our results indicate that model sensitivity to aerosols significantly affects the prediction of amounts and spatial distribution of precipitation, which are critical to mitigate the impacts of extreme precipitation in densely populated urban areas. Notably, operational forecasting models often exclude aerosol dynamics and atmospheric chemistry due to the high computational costs. However, our findings suggest that neglecting aerosol dynamics can lead to underestimations of precipitation by several millimeters over DFW, and potentially miss the detection of extreme events. Future studies using a larger ensemble of deep convection events could provide more robust insights on the role of aerosols in impacting the occurrence and evolution of such events.

Further, we speculate that the interaction between extreme UHI (Hawkins et al. 2004), anthropogenic aerosols, and background biogenic aerosols may impact precipitation differently depending on the background climate. For example, in arid regions, the UHI effect is typically more pronounced at night. However, unlike humid regions, where urban areas are generally warmer throughout the day, arid cities can sometimes experience a negative UHI effect, known as the urban cool island (UCI) (Yang et al. 2017). In a city like Phoenix, AZ, urbanization has been shown to predominantly suppress precipitation (Yang et al. 2016). Further investigation is needed to understand the underlying mechanisms, and the proposed method could help identify the key drivers responsible for these effects.

Our study also highlights the need for further research to better understand the combined effects of UHI, urban expansion, and emissions on local and regional atmospheric processes, particularly in the context of a warming climate and different Shared Socioeconomic Pathways. While some studies suggest that the UHI’s influence on precipitation patterns may weaken in a warmer climate (Saharia et al. 2017), the complex, non-linear feedbacks between potential reductions in anthropogenic emissions and continued urban expansion remain a critical and uncertain issue.

In particular, the interaction between anthropogenic and biogenic aerosols, which influence cloud lifecycles in coastal cities, remains poorly understood (González et al. 2021). Sensitivity studies under different emissions scenarios are essential to addressing these uncertainties. Consistent with previous research (Han et al. 2012; Zhang et al. 2018), our findings underscore the significant impact of anthropogenic aerosols on extreme precipitation events. Future studies should assess how potential emission reductions, such as the widespread adoption of electric vehicles, might mitigate these effects.
